# Bugs Bugging the Body and the Brain: A Case of a Bed Bug Infestation Progressing to Delusions of Parasitosis Treated With Clozapine

**DOI:** 10.7759/cureus.80110

**Published:** 2025-03-05

**Authors:** Daniel Hahn, Dylan Miller, Jasmine Fung, Hansl Mo, Suzanne Lind

**Affiliations:** 1 Psychiatry, Touro College of Osteopathic Medicine, Touro University, New York, USA; 2 Psychiatry, St. Mary's General Hospital, Passaic, USA

**Keywords:** bed bug infestation, clozapine, delusional parasitosis, delusions of parasitosis, ekbom syndrome

## Abstract

Delusions of parasitosis, also known as delusional parasitosis or Ekbom syndrome, is a rare psychiatric disorder characterized by a fixed belief in a bug infestation, despite the absence of clinical evidence. It often co-occurs with other psychiatric conditions such as depression, anxiety, and schizophrenia. In this case, a 50-year-old man with bipolar-type schizoaffective disorder developed delusional parasitosis after a bed bug infestation in his group home. His inadequate adherence to prescribed medications likely exacerbated his symptoms. Treatment involved the consistent administration of clozapine and valproic acid, with dosage adjustments and close monitoring by a psychiatric team. Notably, clozapine's successful role in this case is unique, as delusional parasitosis is typically treated with second-generation atypical antipsychotics.

## Introduction

Delusional parasitosis, also known as Ekbom syndrome, is a rare psychiatric disorder characterized by a persistent belief in a parasite or insect infestation, despite a lack of supporting evidence [1]. It typically presents with hallucinations, itching, and sensations of biting or crawling on the skin [2]. A hallmark of delusional parasitosis is the “matchbox sign,” in which patients bring items such as insects, flakes of skin, hair, or pieces of fabric as evidence of their infestation [1,2]. Affected individuals may exhibit distress through behaviors of compulsive scratching, excessive cleaning, and self-mutilation in attempts to remove the perceived parasites [2]. In severe cases, the condition can become a psychiatric emergency when patients exhibit risky behaviors in response to the delusion, such as setting fire to belongings, using toxic substances for self-treatment, or even attempting suicide [3,4]. Additionally, shared delusional parasitosis can occur, with variants like “folie a deux” for two individuals, “folie à famille” for an entire family, or delusional parasitosis by proxy, where the patient believes others are also infested [1,5]. 

Delusional parasitosis can present as either chronic or acute: the chronic form is typically seen in primary delusional parasitosis, while acute episodes occur in the secondary form [4]. Primary delusional parasitosis is an isolated condition, while secondary delusional parasitosis arises in conjunction with or as a result of another neurological or psychological disorder [4,6]. Isolating a single cause for delusional parasitosis is challenging, as studies have shown it has been linked to a variety of contributing factors [4]. It often develops in association with underlying psychiatric conditions, such as schizophrenia, bipolar disorder, depression, anxiety, or obsessive-compulsive disorder [4]. More than half of patients with delusional parasitosis have a history of depression, and many experience both conditions concurrently [6]. 

The pathophysiology of delusional parasitosis remains uncertain, though many support the theory of dopaminergic imbalances and striatal dopamine transporter (DAT) dysfunction, a presynaptic plasma membrane protein responsible for maintaining dopamine levels in the inter-synaptic space [1]. Disorders that impair dopamine transporter function, such as Parkinson’s disease, Huntington’s disease, human immunodeficiency virus (HIV) infection, traumatic brain injury, alcoholism, and iron deficiency, may produce similar symptoms or lead to secondary forms of delusional parasitosis [5]. Secondary causes have also been linked to hyperthyroidism, diabetes, and vitamin B12 deficiencies [5,7].

Delusional parasitosis can also result from organic causes, including medication side effects and substance abuse. Medications such as ciprofloxacin, ketoconazole, topiramate, and corticosteroids are associated with delusional parasitosis as a side effect [7]. Moreover, substance abuse and withdrawal can trigger delusional parasitosis, with cocaine users experiencing a specific form of delusional parasitosis known as cocaine formication, or “cocaine bugs” [1]. It is crucial to rule out actual parasitosis before diagnosing delusional parasitosis [1]. A significant number of patients with schizophrenia live in group homes, which are more susceptible to bed bug infestations [8]. These alternative causes of delusional parasitosis, along with the need to exclude actual parasitosis, should be considered in the differential diagnosis. 

Schizoaffective disorder, as seen in this patient, is characterized by schizophrenic-like thinking or bizarre behavior combined with affective states, which may be of the bipolar or depressive type. The bipolar type includes manic or mixed episodes, while the depressive type is limited to major depressive episodes [9]. Controlled studies have shown that a combination of the mood stabilizer valproate for mood symptoms and the antipsychotic clozapine for schizophrenia symptoms is effective in treating schizoaffective disorder, both of which were used in this patient’s treatment regimen [10,11].

In this case, a 50-year-old man with a history of bipolar-type schizoaffective disorder, who had previously lived in a bed bug-infested home, presented to the hospital with delusions of parasitosis. His delusional parasitosis symptoms were successfully treated with clozapine, a medication rarely used for this condition, highlighting the need for further investigation into alternative treatments beyond the standard first-line options.

## Case presentation

A 50-year-old man with a past medical history of bipolar-type schizoaffective disorder for the past six years presented to the emergency department with bed bug bites that were primarily visualized on his arms. The patient has no notable family history of psychiatric illness and has been unemployed for the past six years. He had been residing in a bed bug-infested group home, which most likely contributed to his condition and worsened his psychological distress. The group home was then fumigated, and the patient was relocated to a relative’s house where he resided for a month. It was at this point when he reported the onset of auditory hallucinations, despite being in a clean environment. Upon physical examination, bed bug bites were visualized on the patient’s arm, which served as evidence of actual bed bugs being present at one point.

Soon after, the patient began to express that bugs were present in his head and ears, controlling his brain, and insisting that he not argue with them. He complained of tactile hallucinations at this point, such as blood loss and draining pus from his ears due to his body being plagued by the bugs. When he was brought to the emergency department again, a Mental Status Examination (MSE) was performed with a psychiatric clinical interview. The patient had a disheveled appearance with psychomotor agitation and fleeting eye contact and was seen pacing around the room. His behavior was superficially cooperative, guarded, and minimizing. His speech was coherent and at a normal rate, but there was poverty of content and increased latency of responses. 

The patient stated that his mood was “okay,” but his affect was observed as labile, withdrawn, and with a blunted range. His thoughts were linear and concrete; however, his thought process was blocked, he was paranoid, and he had delusions of being controlled. The patient was alert and oriented to person, place, and time. He had grossly intact attention, concentration, cognition, and memory. He exhibited limited insight, minimized his current illness, and had poor judgment. On physical examination, the patient appeared disheveled and anxious with bilateral hyperemic eyes. During neurologic examination, the patient presented with tremulous, repetitive movements of both hands. 

For the past five years, the patient was previously prescribed clozapine 25 mg orally twice daily for schizoaffective disorder, valproic acid 500 mg orally twice daily for mood stability, benztropine 1 mg orally twice daily for extrapyramidal symptoms, trazodone 100 mg orally once daily for insomnia, and paliperidone palmitate 234 mg intramuscularly every month for schizoaffective disorder. The patient insisted that he was compliant with his psychiatric medications, but a review of his medical records revealed that he had not renewed his psychiatric medications and was lost to follow-up on his monthly paliperidone injection for the previous two months. 

On admission to the psychiatric department, clozapine was increased from 25 to 50 mg, and his valproic acid was reinitiated at 500 mg, both administered orally bidaily. The patient’s cooperability improved; however, his blunted and withdrawn affect remain unchanged. After increasing the clozapine dosage to 50 mg orally twice daily, the patient’s auditory and visual hallucinations were minimized. During his admission, the patient’s medication regimen was slowly titrated to 75 mg of clozapine orally twice daily and 750 mg of valproic acid orally twice daily. On day 7, the patient expressed resolution of his auditory hallucinations. He was admitted for three weeks, with hematologic testing conducted weekly to monitor for side effects of his prescribed medications. No new hematologic adverse effects were noted due to the medication (Table 1).

**Table 1:** Hematological laboratory values after the re-initiation of medications in the psychiatry inpatient care WBC: white blood cell, RBC: red blood count, MCV: mean corpuscular volume, MCH: mean corpuscular hemoglobin. MCHC: mean corpuscular hemoglobin concentration, RDW: red cell distribution width, MPV: mean platelet volume, nRBC: nucleated red blood cell

	Emergency department (day 1)	Psychiatry inpatient (day 2)	Psychiatry inpatient (day 10)	Psychiatry inpatient (day 14)
WBC	5.5	5	5.9	5
RBC	4.33	4.06	4.11	4.62
Hemoglobin	14.2	13.4	13.3	15.1
Hematocrit	42.2	39.4	39.5	44.4
MCV	97.6	96.9	96	96.1
MCH	32.8	32.9	32.3	32.7
MCHC	33.7	33	33.6	34
RDW	14.4	14.4	14	14
Platelets	129	133	191	186
MPV	9.6	9.5	9.3	9.4
Neutrophils (relative)	61.7	46.8	64.9	47
Lymphocytes (relative)	29.1	41	23.1	42
Monocytes (relative)	8.2	9.8	10.1	8
Eosinophils (relative)	0.6	1.7	1.4	2
Neutrophils (absolute)	3.4	2.3	3.8	2.4
Lymphocytes (absolute)	1.6	2	1.4	2.1
Monocytes (absolute)	0.4	0.5	0.6	
Eosinophils (absolute)	0	0.1	0.1	0.1
Basophils (absolute)	0	0	0	
nRBC	0	0	0	0.01

Once stabilized, the patient was discharged at this final dosage. The patient’s family was consulted, and individual and group therapies were included in the treatment plan. Group therapies were conducted weekly to monthly, focusing on community support and socialization, life skills, stress management, and recreation. The sessions have been ongoing since the patient was discharged, and the patient has been compliant in attending these therapy groups. 

After being discharged, the patient continued the prescribed medications at his family’s home, to which his mood, appetite, and sleep stabilized. The patient continued to have monthly follow-ups in both the outpatient psychiatric clinics and the group home setting. On follow-up, the patient reported no additional delusional parasitosis symptoms; an absence of auditory, visual, or tactile hallucinations; no suicide ideation; and no acute episodes of schizoaffective symptoms. With adherence to his medications and attending support groups, the patient’s overall affect improved. He continues to experience improvements in his quality of life, including better eating and sleeping. The patient reports that the medications are still helpful. 

## Discussion

Delusional parasitosis is a rare psychiatric disorder, with an incidence rate of 1.0-1.9 per 100,000 person-years in the United States [1,7]. The average age of diagnosis is 61 years old, and it is most commonly diagnosed in middle-aged women [1,2,5]. Symptoms include delusions and tactile hallucinations of bugs, such as the sensation of biting, crawling, tingling, movement under the skin, and pain [5]. 

Diagnosing the syndrome requires a multidisciplinary approach, as patients usually consult dermatologists and infectious disease specialists first to evaluate the bites and to rule out underlying pathologies [1,4,5]. It is only investigated as a psychiatric condition later in its progression after it meets the criteria of conviction of an infestation despite any evidence and abnormal cutaneous sensations that contribute to their belief [1]. Diagnosis can be challenging because many patients refuse psychiatric treatment due to their firm belief in an actual infestation [5]. Self-treatment is common, which can cause complications with non-medication-related injuries [1,3,4,5]. However, it is reported that only 2% of bed bug infestations progress to symptoms of delusional parasitosis [12]. Oftentimes, these patients will seek multiple clinicians to validate their delusions instead of treating the underlying pathology [4,7].

Delusional parasitosis is typically a complex condition to treat, as patients suffering from it tend to be noncompliant with their medications [7]. While the efficacy between first- and second-generation antipsychotics and the consensus of dosage remains a much-needed area of research, currently, second-generation atypical antipsychotics, such as risperidone or olanzapine, are considered first-line treatments of delusional parasitosis [5]. These medications improve altered dopamine transmission via a blockage of the dopamine D2/D3 receptors in the ventral striatal regions. This diminished symptomatology of delusional parasitosis, as elevated dopamine levels at the synapse activate dopaminergic neurons in the ventral tegmental area and their projections to the nucleus accumbens, is associated with itching and scratching behaviors [1].

The first widely used antipsychotic for the treatment of delusional parasitosis was pimozide; however, more recently, its popularity as a first-line treatment has diminished due to its concerning side effects, such as prolongation of the QT interval and high-risk extrapyramidal symptoms [1,5,13]. Other atypical antipsychotics with better safety profiles, such as olanzapine, risperidone, paliperidone, aripiprazole, and quetiapine, have been utilized instead [1,5]. Each atypical antipsychotic carries a different risk-benefit profile, such as aripiprazole having a lower metabolic risk, quetiapine having a sedative profile, and risperidone having a moderate, dose-dependent risk of extrapyramidal symptoms (compared to olanzapine) [14]. Freudenmann and Lepping, in a critical literature review of the outcome and efficacy of second-generation antipsychotics for delusional parasitosis, determined that risperidone and olanzapine were the most commonly prescribed, positively impacting 69% and 72% of patients, respectively [13]. A three-case series report by Meehan et al. demonstrated the effectiveness of olanzapine in treating delusional parasitosis and suggested it be considered a first-line therapy [15]; however, it carries metabolic adverse effects, such as weight gain, diabetes, and dyslipidemia, which may make it unsuitable for certain patients [14,15]. It is understood that olanzapine has dopaminergic and serotonergic antagonistic selectivities for mesolimbic and mesocortical over striatal dopamine tracts, therefore minimizing its extrapyramidal adverse effects [15,16]. Furthermore, olanzapine’s 5-HTA2 antagonism enhances the release of dopamine from the nigrostriatal pathway [16]. However, olanzapine’s metabolic side effects are likely mediated by histaminergic (H1) and muscarinic (M3) receptor blockage [16].

A systematic review by Lu et al. comparing first-generation and second-generation antipsychotics for primary delusional infestation found that while both classes had comparable efficacy, patients using second-generation antipsychotics experienced higher rates of complete remission and lower rates of treatment failure, likely due to their more favorable side effect profiles [17]. First-generation antipsychotics are known to produce more severe extrapyramidal side effects, such as parkinsonism, acute dystonia, akathisia, and tardive dyskinesia, which can decrease the quality of life and, therefore, compliance for these patients. Lu et al. found that risperidone is the most widely studied second-generation antipsychotics, followed by olanzapine, which had a higher complete remission rate, lower non-effectiveness rate, and a higher incidence of metabolic side effects, such as weight gain [17]. Interestingly, aripiprazole has the highest complete remission rate compared to risperidone and olanzapine [17]. Further, aripiprazole also acts as a partial dopamine agonist, making it a helpful adjunct in treating depression, which is known to be a common comorbidity in patients with delusional parasitosis [18]. This systematic review also looked at the use of selective serotonin reuptake inhibitors, specifically fluoxetine or citalopram, as a treatment for delusional infestation [17]. While the study’s sample size was a small population with comorbid depression, anxiety, and trichotillomania, it found that patients had complete remission of delusional parasitosis symptoms with selective serotonin reuptake inhibitors [17]. There is still no strong evidence suggesting the efficacy and use of one of these specific medications over the other [13,17].

Clozapine, an atypical antipsychotic, has not been well-documented in the literature as a primary treatment for delusional parasitosis. Clozapine is not the first-line treatment for schizophrenia due to its wide range of adverse side effects. While it has been studied to be effective in the treatment of schizophrenia, there have been reported deaths in the United States due to clozapine-induced agranulocytosis. Other notable side effects include myocarditis, metabolic syndrome, seizures, excessive salivation, pulmonary embolism, constipation, and neuroleptic malignant syndrome [19]. One case report discussed the successful treatment of delusional parasitosis with clozapine and electroconvulsive therapy (ECT) after limited improvement with oral psychotropics [20]. After attempted treatment with pimozide, risperidone, and olanzapine, with limited clinical improvement, a clozapine regimen was initiated at 25 mg twice daily. This dosage was slowly increased to 600 mg twice daily over multiple weeks, while a trial of electroconvulsive therapy was given simultaneously. After a total of 12 bitemporal ECT sessions, administered three times a week, gradual improvement in aggression, paranoia, and delusion symptoms was seen [20].

This is a unique case both in its pathological development and treatment. It is rare for patients with bed bug infestations to progress to delusional parasitosis [12]. In this patient, a case of actual infestation of bed bugs progressed to delusional parasitosis. Additionally, delusional parasitosis is typically treated with second-generation antipsychotics such as risperidone and olanzapine. However, for this patient, clozapine was used to treat delusional parasitosis without ECT. This is an area with limited research that has not been thoroughly explored, making this one of the first cases to display clozapine’s effective use in treating delusional parasitosis.

## Conclusions

This case highlights the importance of diagnosing primary versus secondary causes of delusional parasitosis, prescribing a tailored treatment regimen, and patient compliance with therapy. Patients with psychiatric conditions should be closely monitored for delusional parasitosis. Physicians should be keenly aware that patients with schizophrenia and schizoaffective disorder who have been exposed to actual pests can present with delusional parasitosis as a compounding manifestation of both their medical issues with bites and their psychiatric issues.

While second-generation antipsychotics are the first-line treatment for delusional parasitosis, their adverse side effects are still of concern and warrant further investigation into alternative treatment options. For patients who present with refractory delusional parasitosis, where other medications are not working, clozapine should be considered a treatment. Moreover, for patients already on a clozapine regimen for a comorbidity who present with delusional parasitosis, adjusting the clozapine dosage may be an effective initial treatment. Clozapine is an atypical antipsychotic with several possible severe side effects, including metabolic, hematological, or neurological side effects. Long-term effects such as agranulocytosis may pose a risk to patients; however, in treatment-resistant delusional parasitosis, clozapine may still be a beneficial treatment regimen. In this case, clozapine and valproic acid were used to successfully treat the patient despite not being suggested first-line treatments.
